# PEARS: A Web Tool for Fitting Time-Resolved Photoluminescence Decays of Perovskite Materials

**DOI:** 10.1021/acs.jcim.3c00217

**Published:** 2023-07-18

**Authors:** Emmanuel V. Péan, Matthew L. Davies

**Affiliations:** † SPECIFIC IKC, Materials Research Centre, College of Engineering, Swansea University Bay Campus, Fabian Way SA1 8EN, Swansea, U.K.; ‡ School of Chemistry and Physics, University of KwaZulu-Natal, Durban 03209, RSA

## Abstract

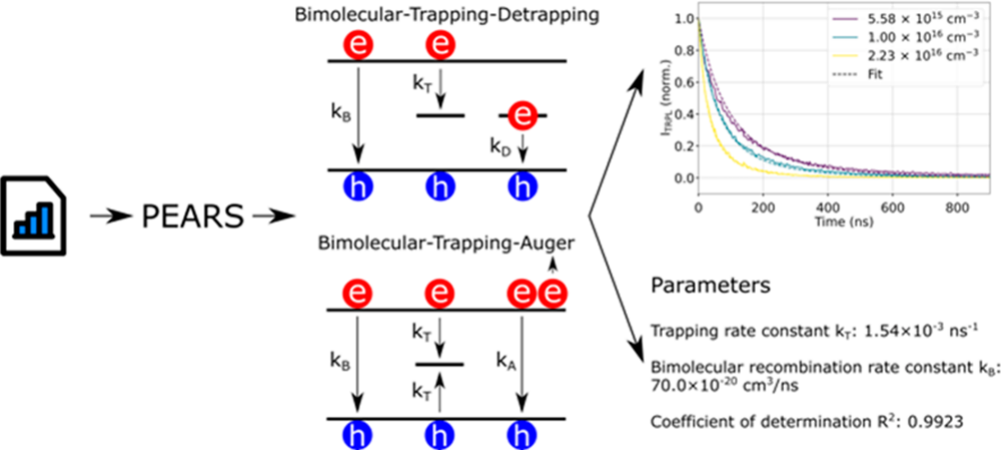

Time-resolved photoluminescence (TRPL) is a powerful tool to investigate charge carrier recombination processes in emissive materials. Perovskite materials are extremely promising for applications in solar cells; however, the interpretation of their TRPL is arduous due to the complicated nature of the recombination processes occurring in these materials. We present here the PErovskite cArrier Recombination Simulator (PEARS) web tool for effortlessly and quickly fitting TRPL of perovskite materials using advanced charge carrier recombination models, allowing for the extraction of recombination rate constants and trap state concentration. PEARS is flexible and can adapt to different situations, by ignoring recombination processes or fixing known parameters (e.g., the doping concentration). The tool is publicly available at https://pears-tool.herokuapp.com.

## 1 Introduction

Thanks to their high absorption coefficient and ideal band gap,^1^ perovskite materials are excellent candidates for the next generation of solar cells with an impressive power conversion efficiency of 25.7% certified.^2^ However, the development of perovskite-based solar cells has been impeded by obstacles such as the degradation of the perovskite layer by oxygen and moisture under illumination.^3−10^ Furthermore, (photo) excited carriers have been shown to be responsible for the intrinsic instability of the perovskite structure through the formation of iodine, the photocatalysis of iodine, and the photolysis of lead iodide among others.^7,11^

Photoluminescence (PL) spectroscopy measures photoexcited carrier radiative recombinations and is thus a great tool for studying charge carrier processes in emissive materials and solar cells. While steady-state PL consists of recording the emission intensity of a sample under continuous excitation, time-resolved PL (TRPL) is a measurement of emission intensity over time after a short excitation pulse, typically fs-ps. TRPL allows one to study the transient phenomenon happening after excitation, including charge carrier recombinations, energy transfer, and, in the case of thin-film materials containing multiple layers (e.g., solar cells), injection processes.^12−15^ However, the measurement and interpretation of TRPL decay curves of perovskites are usually difficult due to the complex recombination processes happening in these materials.^16^ Like many other semiconductors, due to their low exciton binding energy, direct electron–hole recombinations are bimolecular in lead halide perovskites, leading to a dependency of the carrier concentrations and TRPL on the excitation fluence.^16^ This causes TRPL decays to be dependent not only on the intrinsic properties of the sample studied (e.g., trap state concentration) but also on its extrinsic properties (thickness) and the measurement parameters (laser fluence). While this behavior is not unique to perovskites, their sensitivity to external stimuli, and resulting transient properties, made them somewhat difficult samples to accurately retrieve quantitative information from. Consequently, TRPL of perovskite materials is often used as an indicative tool in the literature, sometimes analyzed using a multiexponential decay model which does not account for the nonexcitonic nature of these materials and, therefore, may lead to misinterpretation of the results.^16^

Here, we present the PErovskite cArrier Recombination Simulator (PEARS) web tool allowing effortless and quick fit of the TRPL of perovskite thin films using proven and tried physical models. We first discuss the two charge carrier recombination models available within the tool as well as the method used to fit the TRPL data. Analysis of the fitting results through the calculation of the different process contributions as well as the carrier accumulation is then discussed. Finally, we go through a step-by-step example of using the tool to analyze perovskite TRPL data.

## 2 Models and Computational Details

The two charge carrier recombination models available within PEARS are extensively discussed in our previous paper^16^ and only differ by the addition of Auger recombination to one of the models. PEARS is written in Python^17^ using the SciPy,^18^ NumPy,^19^ and Pandas^20^ packages for data processing and the Streamlit, Plotly,^21^ and HiPlot^22^ packages for the graphical user interface and data plotting.

### 2.1 Models

**Figure 1:**
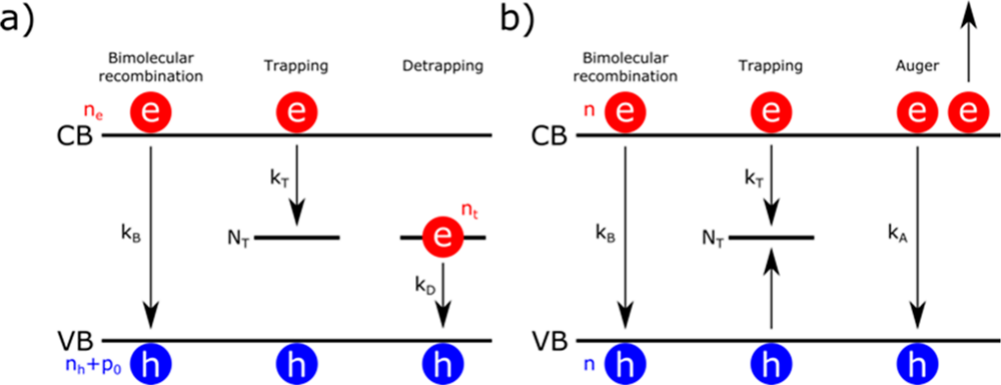
Schematic representation of the **a)** Bimolecular-Trapping-Detrapping and b) Bimolecular-Trapping-Auger models. Excited electrons (concentration *n*_e_ or *n*) in the conduction band (CB) and holes (concentration *n*_h_ with *p*_0_ doping or *n*) in the valence band (VB) bimolecularly recombine at rate constants *k*_B_. In **a)** electrons get trapped in trap states (concentration *N*_T_) at the rate constant *k*_T_. Trapped electrons (concentration *n*_t_) then detrap back to the VB at the rate constant *k*_D_. In **b)**, the trapped electron concentration is assumed to be negligible, and electrons and holes get trapped in trap states at rate constant *k*_T_. Finally, Auger recombinations happen at the rate constant *k*_A_.

PEARS allows one to choose between two charge carrier recombination models that have been extensively used in the literature:^12,15,16,23^ the Bimolecular-Trapping-Detrapping (BTD) and Bimolecular-Trapping-Auger (BTA, also known as the ABC or *k*_1_*k*_2_*k*_3_ models in the literature) models. Both models consider direct bimolecular recombination between electrons and holes but differ in their treatment of trap-mediated recombination (Figure 1).

Within the BTD model, it is assumed that the system studied is P-type with electron trapping, although the opposite (N-type with hole trapping) is also valid by simply switching the notation. Trapping is considered as a bimolecular process between the free electrons and the available trap states, and detrapping is considered as a bimolecular process between the trapped electrons and the free holes^12,16^ (Figure 1a). The rate equations of the BTD model giving the variations of the carrier concentrations over a small period of time are^12,16^
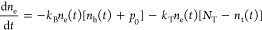
1
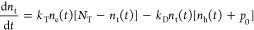
2
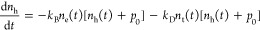
3where *n*_e_ is the photoexcited electron concentration, *n*_n_ is the photoexcited hole concentration, *n*_t_ is the trapped electron concentration, *k*_B_ is the bimolecular recombination rate constant, *k*_T_ is the trapping rate constant, *k*_D_ is the detrapping rate constant, *p*_0_ is the dark hole concentration, and *N*_T_ is the trap state concentration. These equations are solved using the fact that the same concentrations of electrons and holes (*N*_0_) are photoexcited just after the excitation pulse while the trap states are assumed empty.
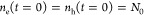
4
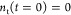
5

The photoexcited concentration *N*_0_ generated by an excitation pulse of energy *I*_0_ (in photons/cm^2^) in a thin film of thickness *D* and absorptance *A* can be approximated as^16^
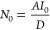
6

Finally, the TRPL intensity is proportional to the radiative bimolecular recombination rate.^16^
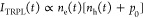
7

The BTA model is a simplification of the BTD model and assumes that the trap states remain mostly empty, thus allowing trapping and detrapping to be considered monomolecular processes, and that the electron and hole concentrations are identical at all times (i.e., *n*_e_(*t*) = *n*_h_(*t*) = *n*(*t*)) (Figure 1b). This allows one to simplify the above rate equations, leading to^16^
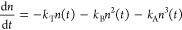
8where *k*_T_ is the monomolecular trapping rate constant, *k*_B_ is the bimolecular rate constant, and *k*_A_ is the Auger recombination rate constant. The initial condition used to solve this differential equation is
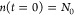
9

The TRPL intensity is calculated from the carrier concentrations using^16^
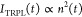
10

Within PEARS, the user can select the model to fit the data. The model used should depend on the behavior of the experimental TRPL decays as previously discussed in ref (16). We note that, to date, these models have been used on a specific subset of perovskite materials, such as methylammonium lead iodide and methylammonium lead iodide-chloride, and therefore may not be suitable for more complex materials such as mixed-halide perovskites.

**Figure 2:**
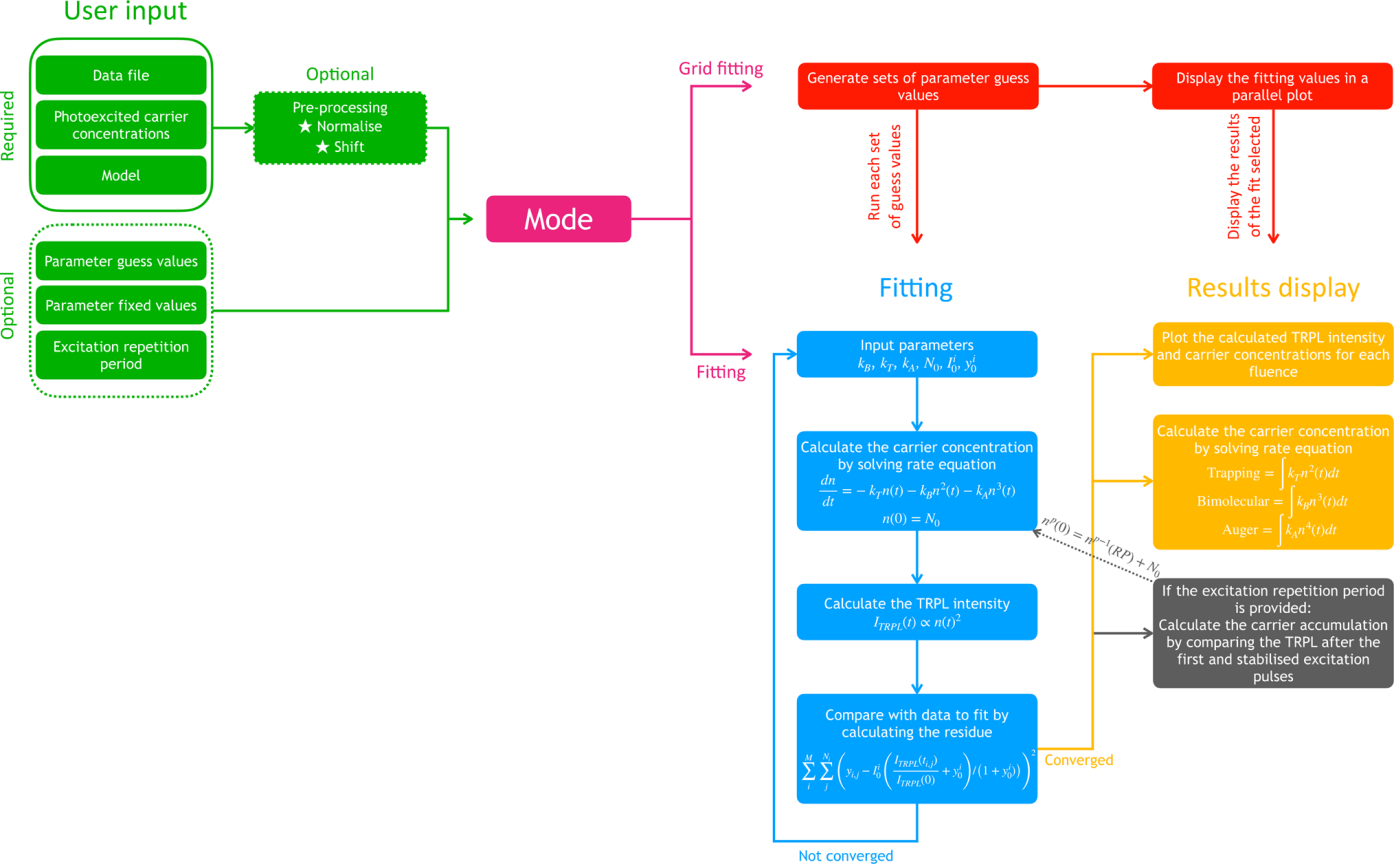
Schematic representation of the PEARS tool highlighting the processes in the case where the BTA model is used.

The tool is schematically represented in Figure 2 in the case where the BTA model is used. We now discuss the method employed to fit the data using the above models.

### 2.2 Fitting

Fitting of multiple experimental TRPL curves is carried out using a global least-squares optimization. For a data set containing *M* curves, each curve *i* containing *N*_*i*_ data points, the optimization residue SS_res_ is
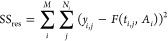
11where *y*_*i,j*_ is the experimental TRPL intensity at time *t*_*i,j*_ of point *j* of curve *i*. *A*_*i*_ are the model parameters associated with curve *i*, and *F* is the fitting model given by
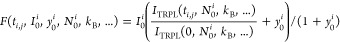
12where *I*_0_ is an intensity factor, *y*_0_ is an intensity offset, and *I*_TRPL_ is calculated using one of the models above. The intensity factor can be fixed (by default) or left free during the least-squares optimization, which may improve the accuracy of the parameters retrieved (Figure S1). The factor 1/(1 + *y*_0_^*i*^) allows normalization of the intensity to *I*_0_^*i*^ after the intensity offset has been added to the normalized TRPL. The quality of the fit is estimated using the coefficient of determination *R*^2^
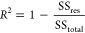
13where SS_total_ is defined as the sum of the squared difference between each point and the average of all curves *y̅*.
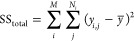
14

The least-squares optimization is solved using a trust region reflective algorithm as implemented by the SciPy package.^18^ The optimization is run using guess values that the user can set. By default, values from the literature are used.^15,16^ It is also possible to set fixed values for certain parameters, in which case these parameters are not optimized during the fitting process. This allows one to ignore certain processes (e.g., Auger recombinations) by setting the associated recombination rate constant (e.g., *k*_A_) to zero. After a successful fit, PEARS calculates the contribution of each recombination process to the TRPL variations over time.

### 2.3 Process Contributions

The contributions of each process (e.g., bimolecular recombination) to the TRPL variations over time (i.e., how much each process affects the TRPL decay shape) are calculated from the parameter values obtained from the fit using eqs 24 and 25 from ref (16) (Figure 2). For the BTA model, the trapping *T*, bimolecular *B*, and Auger *A* contributions are
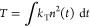
15
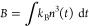
16
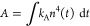
17

Within this model, a low contribution can indicate that the associated parameter is inaccurately retrieved due to too high or too low fluences used to measure the TRPL.^16^ For example, a low bimolecular contribution suggests that *k*_B_ is not accurately retrieved by the fit and that higher excitation fluences should be used to measure the TRPL. PEARS automatically highlights contributions below 10% and gives subsequent recommendations. It is important to note that this threshold is dependent upon the signal/noise ratio of the data and therefore it is not an absolute value.^16^ For the BTD model the bimolecular, trapping, and detrapping *D* contributions are
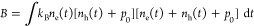
18
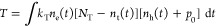
19
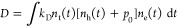
20

Within the BTD model, a non-negligible contribution does not guarantee that the associated parameter values are accurately retrieved. This is due to the higher complexity of this model, which can result in very similar TRPL decays from different sets of parameters.^16^ This model thus requires grid fitting to find any potential local minima, as described below.

### 2.4 Grid Fitting

Grid fitting is the secondary operation mode of PEARS and consists of running multiple sets of guess values to ensure that only a single fit to the data exists (Figure 2). The grid is generated from a list of supplied guess values (e.g., *k*_B_: 10^–20^, 10^–19^ and *k*_T_: 10^–3^, 10^–2^ yields 4 sets of guess values: (10^–20^, 10^–3^), (10^–20^, 10^–2^), (10^–19^, 10^–3^), and (10^–19^, 10^–2^)). A list of guess values is provided by default by the tool; however, the user can easily modify them. Note that in the case of the BTD model only sets of guess values satisfying *k*_T_ > *k*_B_ and *k*_T_ > *k*_D_ are considered to reduce the computational cost, as the trapping rate constant is expected to be higher than the bimolecular and detrapping rate constants in perovskite materials. Fitting is then carried out using each set of guess values, as schematically represented in Figure S2.

### 2.5 Carrier Accumulation

Carrier accumulation happens when an excitation pulse occurs before all photoexcited carriers have recombined.^16^ The resulting increased carrier concentration can affect the TRPL decay shape and therefore should be avoided during experimental measurements by choosing an adequate excitation repetition period. PEARS simulates only the first excitation pulse and therefore does not consider carrier accumulation during fitting due to the high computational cost required to do so (which can multiply the calculation time from 2 to 100 times), and it is therefore assumed that the TRPL decays provided by the user are unaffected by carrier accumulation. However, the effect of carrier accumulation can be estimated from the values retrieved from fitting as a self-consistency check if the excitation repetition period is provided. Carrier accumulation (CA) is calculated as the maximum difference between the calculated normalized TRPL after the first (*p* = 1) and stabilized (*p* = *s*) pulses
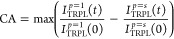
21where the stabilized pulse is defined as the pulse when the carrier concentrations vary by less than 10^–3^% of the photoexcited concentration between two consecutive pulses.
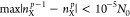
22

If the fitting predicts a high carrier accumulation, then the solution is not self-consistent and the values obtained from the fit are likely incorrect.

### 2.6 Sample Manufacturing and TRPL Measurements

To showcase the abilities of PEARS, we fitted the TRPL of an MAPI thin film measured with 3 different excitation fluences.

The MAPI perovskite precursor solution was prepared in an ambient atmosphere from a 1.25 M/1.25 M solution of PbI_2_ (*Sigma-Aldrich*, 99%) and MAI (*Dyesol*) in 4:1 (v/v) dimethylformamide (DMF) (*Sigma-Aldrich*, ≥99.8%)/dimethyl sulfoxide (DMSO) (*Sigma-Aldrich*, ≥99.9%), heated at 60 °C until dissolution. Glass substrates were cleaned using *Hellmanex III*, acetone, and isopropanol consecutively. The substrates were cleaned with an O_2_ plasma for 5 min just before spin-coating to improve the spreading of the solution. 100 μL of perovskite solution was spin-coated at 4500 rpm (4500 rpm/s acceleration) in an ambient atmosphere for 30 s. 200 μL of ethyl acetate was deposited while spinning after 15 s as an antisolvent treatment to promote crystallization. The film was then annealed at 100 °C for 10 min. In order to hinder degradation by moisture during the measurements, a thin poly(methyl methacrylate) (PMMA) layer was spin-coated on top of the perovskite layer at 4500 rpm (4500 rpm/s acceleration) for 30 s from a 1.0 M solution of PMMA dissolved in toluene. Finally, the film was annealed at 70 °C for 30 min.

The TRPL was measured using an *Edinburgh Instruments Lifespec 2* TCSPC operating in reverse mode and an EPL405 laser (*λ*_exc_ = 405 nm). The sample emission was measured at 770 nm with an emission slit width of 17.5 nm and a laser repetition period of 1 μs.

Absorptance was measured using a *PerkinElmer Lambda 750* UV/vis spectrophotometer coupled to a 100 mm integrating sphere. The film thickness was measured with a *Dektak 6M* profilometer.

## 3 Case Study

We now go through a step-by-step example of using PEARS to fit the TRPL data.

**Figure 3:**
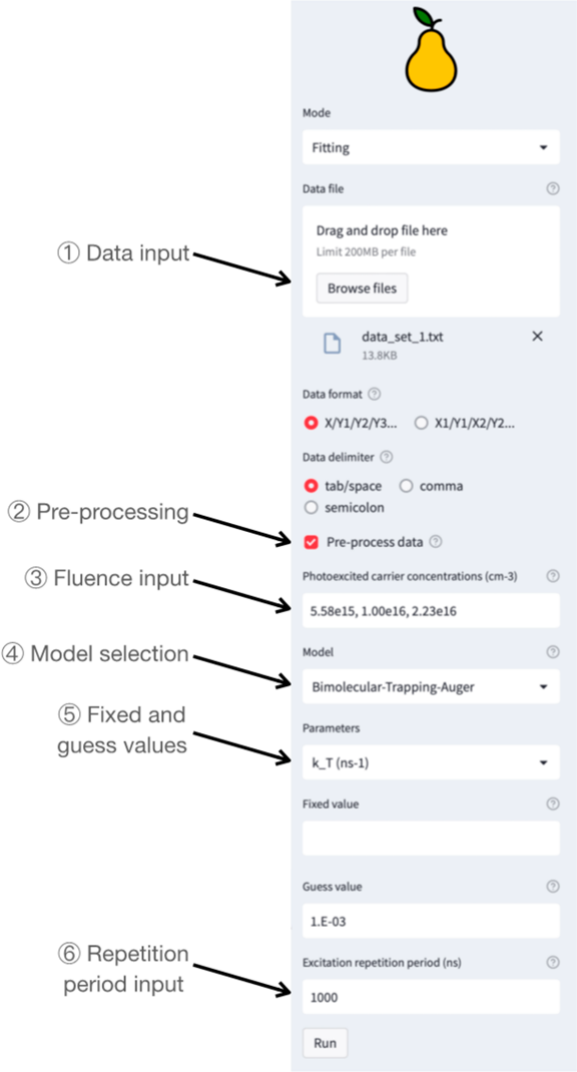
Screenshot of the results display after fitting.

**Figure 4:**
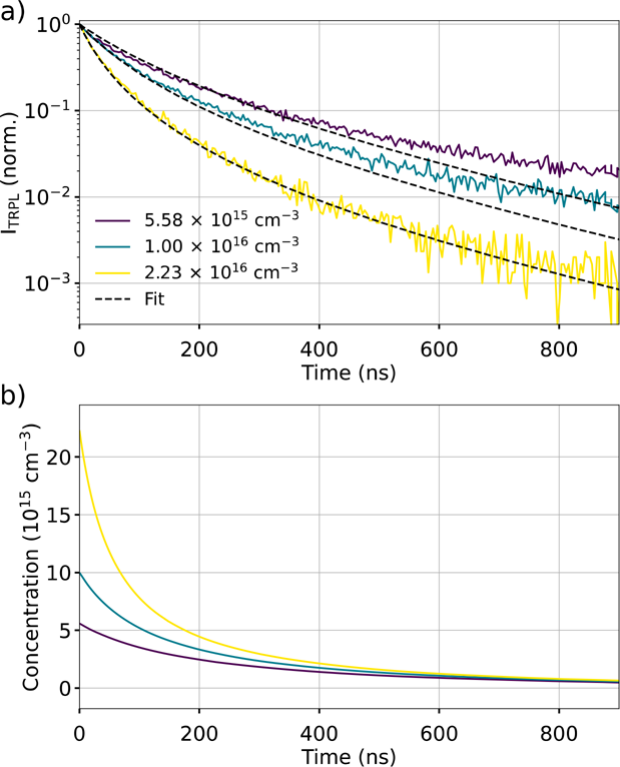
**a)** Fitting result and **b)** associated carrier concentration.

A wide variety of data files can be uploaded. The data delimiter can be chosen among space/tab, comma, or semicolon. Each column should correspond to either the time (X, in nanoseconds) or the TRPL intensity (Y). Two data formats can be read: X/Y1/Y2/Y3/... where the first column corresponds to the time and others correspond to the TRPL intensity, and X1/Y1/X2/Y2/... where the time and intensity columns alternate. For this example, we use “Data set 1” provided online in the “Getting started” section. This file contains TRPL decays of an MAPI thin film measured at 3 different excitation fluences (in order: 5.58 × 10^15^, 1.00 × 10^16^, and 2.23 × 10^16^ cm^–3^). ① Data are uploaded in the sidebar (Figure 3). Since the first column of the data is the time and the other columns are the intensity of each TRPL decay, the data format “X/Y1/Y2/Y3...” is selected. The data are delimited with tabs, and the corresponding “Data delimiter” should be selected. Once the data are correctly loaded, they are displayed graphically. ② Since the maximum intensity of the TRPL decays is not at *t* = 0, the “preprocess data” checkbox is checked. This option offers a quick way to shift the *x*-axis data to zero with respect to each decay’s maximum point and to normalize the intensity. ③ We then input the carrier concentrations photoexcited by 1 excitation pulse for each decay separated with commas (eq 6). ④ Given the simple behavior of the TRPL, we choose to use the BTA model.^16^ ⑤ The value of each parameter of the model can be either fixed or the guess value can be changed. By default, Auger recombinations are ignored as they tend to be non-negligible only at very high excitation fluence, and hence *k*_A_ is fixed to zero. ⑥ Optionally, we set a 1 μs excitation repetition period to estimate the carrier accumulation (this can also be done after the data are fitted). After pressing the “Run” button, PEARS displays the fitting results, including the raw data and the fitted curves (Figure 4), the value of each fitted parameter, the carrier concentrations, and the process contributions at each excitation fluence (Figure S3). In this example, the fit closely matches the input data, with a high coefficient of determination of *R*^2^ = 0.992. A trapping rate constant of *k*_T_ = 1.54 × 10^–3^ ns^–1^ and a bimolecular rate constant of *k*_B_ = 70.0 × 10^–20^ cm^3^/ns are obtained. The maximum trapping and bimolecular contributions are, respectively, 39% and 85%, suggesting that *k*_T_ and *k*_B_ have been accurately retrieved. A maximum of 1.5% difference between the TRPL calculated after the first and stabilized pulse is predicted, thus indicating negligible carrier accumulation and that this solution is self-consistent.

For the second example, we used the BTD model. Running the fitting optimization yields a very good fit with an *R*^2^ value of 0.997. However, due to the complexity of the BTD model, it is imperative to ensure that there are not multiple solutions that can fit the data. Using the “Grid fitting” mode at the top of the sidebar, the fitting optimization is run for multiple sets of guess values, and their optimized parameters are then displayed in an interactive parallel plot (Figure S4). Specific solutions can be selected using the interactive parallel plot, and the details of the first fit of the selection are displayed below it. For this example, multiple solutions of the fit are obtained, all with good *R*^2^ values which do not allow determination of the correct solution physically representing the sample. In this case, further analysis of the sample would be required to determine the value of specific parameters of the model, thus helping to narrow down the correct solution. It is important to note that although this method can prove that multiple solutions to the fitting problem exist for a given data set, it cannot absolutely prove that a unique solution exists.

## 4 Conclusion

We presented here PEARS (PErovskite cArrier Recombination Simulator), a free web tool to effortlessly fit the time-resolved photoluminescence (TRPL) of perovskite materials. PEARS is based on two models widely used in the literature to simulate charge carrier recombinations in nonexcitonic perovskite materials. This tool also offers an analysis of the fit results, through the calculation of the carrier concentrations, the recombination process contributions, as well as carrier accumulation. We showed in 2 examples how to use PEARS using data available within the tool. A first step-by-step example demonstrated the use of the Bimolecular-Trapping-Auger model and how the validity of the recombination rate constants retrieved can be estimated. A second example showed the use of the more complex Bimolecular-Trapping-Detrapping model. However, because of its higher complexity, this model can lead to multiple solutions, which can be analyzed using the “Grid fitting” mode of PEARS. Although PEARS has been developed with perovskite in mind first and foremost, it can also be used for any nonexcitonic materials such as undoped semiconductors. We hope that this new tool will be useful for the community in helping the interpretation of TRPL data. Future updates will include fitting of time-resolved microwave photoconductivity data and include more complex processes, such as charge carrier injection and carrier diffusion.

## Data Availability

PEARS is publicly available at https://pears-tool.herokuapp.com and the base code is available at https://github.com/Emmanuelpean/pears. The example data set used in the Case Study section of this manuscript can be found in the Supporting Information and within the web tool in the “Getting Started” section.

## Supporting Information Available

Comparison of fitting with and without fixed intensity factor, an example of grid fitting, and screenshots of the web tool (PDF)
Example data set used in the Case Study section of this manuscript (TXT)

## References

[ref1] Yang W. S.; Park B.-W.; Jung E. H.; Jeon N. J.; Kim Y. C.; Lee D. U.; Shin S. S.; Seo J.; Kim E. K.; Noh J. H.; Seok S. Il. Iodide Management in Formamidinium-Lead-Halide-Based Perovskite Layers for Efficient Solar Cells. Science (1979) 2017, 356 (6345), 1376–1379. 10.1126/science.aan2301. 28663498

[ref2] Photovoltaic Research, Best Research-Cell Efficiency Chart. National Renewable Energy Laboratory; National Renewable Energy Laboratory. https://www.nrel.gov/pv/cell-efficiency.html (accessed 2023-01-26).

[ref3] Hoke E. T.; Slotcavage D. J.; Dohner E. R.; Bowring A. R.; Karunadasa H. I.; McGehee M. D. Reversible Photo-Induced Trap Formation in Mixed-Halide Hybrid Perovskites for Photovoltaics. Chem. Sci. 2015, 6 (1), 613–617. 10.1039/C4SC03141E. 28706629 PMC5491962

[ref4] Péan E. V.; De Castro C. S.; Davies M. L. Shining a Light on the Photoluminescence Behaviour of Methylammonium Lead Iodide Perovskite: Investigating the Competing Photobrightening and Photodarkening Processes. Mater. Lett. 2019, 243, 191–194. 10.1016/j.matlet.2019.01.103.

[ref5] Brenes R.; Eames C.; Bulović V.; Islam M. S.; Stranks S. D. The Impact of Atmosphere on the Local Luminescence Properties of Metal Halide Perovskite Grains. Adv. Mater. 2018, 30 (15), 1706208 10.1002/adma.201706208. 29512205

[ref6] Mosconi E.; Meggiolaro D.; Snaith H. J.; Stranks S. D.; De Angelis F. Light-Induced Annihilation of Frenkel Defects in Organo-Lead Halide Perovskites. Energy Environ. Sci. 2016, 9 (10), 3180–3187. 10.1039/C6EE01504B.

[ref7] Quitsch W. A.; Dequilettes D. W.; Pfingsten O.; Schmitz A.; Ognjanovic S.; Jariwala S.; Koch S.; Winterer M.; Ginger D. S.; Bacher G. The Role of Excitation Energy in Photobrightening and Photodegradation of Halide Perovskite Thin Films. J. Phys. Chem. Lett. 2018, 9 (8), 2062–2069. 10.1021/acs.jpclett.8b00212. 29624057

[ref8] deQuilettes D. W.; Zhang W.; Burlakov V. M.; Graham D. J.; Leijtens T.; Osherov A.; Bulović V.; Snaith H. J.; Ginger D. S.; Stranks S. D. Photo-Induced Halide Redistribution in Organic-Inorganic Perovskite Films. Nat. Commun. 2016, 7 (May), 11683 10.1038/ncomms11683. 27216703 PMC4890321

[ref9] Tian Y.; Peter M.; Unger E.; Abdellah M.; Zheng K.; Pullerits T.; Yartsev A.; Sundström V.; Scheblykin I. G. Mechanistic Insights into Perovskite Photoluminescence Enhancement: Light Curing with Oxygen Can. Boost Yield Thousandfold. Phys. Chem. Chem. Phys. 2015, 17 (38), 24978–24987. 10.1039/C5CP04410C. 26343504

[ref10] Aristidou N.; Eames C.; Sanchez-Molina I.; Bu X.; Kosco J.; Islam M. S.; Haque S. A. Fast Oxygen Diffusion and Iodide Defects Mediate Oxygen-Induced Degradation of Perovskite Solar Cells. Nat. Commun. 2017, 8, 15218 10.1038/ncomms15218. 28492235 PMC5437277

[ref11] Wang S.; Jiang Y.; Juarez-Perez E. J.; Ono L. K.; Qi Y. Accelerated Degradation of Methylammonium Lead Iodide Perovskites Induced by Exposure to Iodine Vapour. Nat. Energy 2017, 2 (1), 16195 10.1038/nenergy.2016.195.

[ref12] Hutter E. M.; Eperon G. E.; Stranks S. D.; Savenije T. J. Charge Carriers in Planar and Meso-Structured Organic-Inorganic Perovskites: Mobilities, Lifetimes, and Concentrations of Trap States. J. Phys. Chem. Lett. 2015, 6 (15), 3082–3090. 10.1021/acs.jpclett.5b01361. 26267206

[ref13] Savenije T. J.; Guo D.; Caselli V. M.; Hutter E. M. Quantifying Charge-Carrier Mobilities and Recombination Rates in Metal Halide Perovskites from Time-Resolved Microwave Photoconductivity Measurements. Adv. Energy Mater. 2020, 10 (26), 1903788 10.1002/aenm.201903788.

[ref14] Hutter E. M.; Hofman J. J.; Petrus M. L.; Moes M.; Abellón R. D.; Docampo P.; Savenije T. J. Charge Transfer from Methylammonium Lead Iodide Perovskite to Organic Transport Materials: Efficiencies, Transfer Rates, and Interfacial Recombination. Adv. Energy Mater. 2017, 7 (13), 1602349 10.1002/aenm.201602349.

[ref15] Brenes R.; Guo D.; Osherov A.; Noel N. K.; Eames C.; Hutter E. M.; Pathak S. K.; Niroui F.; Friend R. H.; Islam M. S.; Snaith H. J.; Bulović V.; Savenije T. J.; Stranks S. D. Metal Halide Perovskite Polycrystalline Films Exhibiting Properties of Single Crystals. Joule 2017, 1 (1), 155–167. 10.1016/j.joule.2017.08.006.

[ref16] Péan E. V.; Dimitrov S.; De Castro C. S.; Davies M. L. Interpreting Time-Resolved Photoluminescence of Perovskite Materials. Phys. Chem. Chem. Phys. 2020, 22 (48), 28345–28358. 10.1039/D0CP04950F. 33300902

[ref17] Van Rossum G.; Drake F. L. Jr. Python reference manual*;* Centrum voor Wiskunde en Informatica Amsterdam, 1995.

[ref18] Jones E.; Oliphant E.; Peterson P.; . Scipy: Open Source Scientific Tools for Python. http://www.scipy.org/.

[ref19] Van Der Walt S.; Colbert S. C.; Varoquaux G. The NumPy Array: A Structure for Efficient Numerical Computation. Comput. Sci. Eng. 2011, 13 (2), 22–30. 10.1109/MCSE.2011.37.

[ref20] McKinney W. Data Structures for Statistical Computing in Python. In Proceedings of the 9th Python in Science Conference; van der Walt S., Millman J., Eds.; 2010; pp 56–61. 10.25080/Majora-92bf1922-00a.

[ref21] Plotly Technologies, Inc.. Collaborative Data Science*;* Plotly Technologies Inc.: Montreal, QC, 2015. Online at https://plot.ly.

[ref22] Haziza D.; Rapin J.; Synnaeve G. Hiplot, Interactive High-Dimensionality Plots. GitHub repository*;* GitHub, 2020.

[ref23] Herz L. M. Charge-Carrier Dynamics in Organic-Inorganic Metal Halide Perovskites. Annu. Rev. Phys. Chem. 2016, 67 (1), 65–89. 10.1146/annurev-physchem-040215-112222. 26980309

