# Supplementary material for: PEARS: A Web Tool for Fitting Time-Resolved Photoluminescence Decays of Perovskite Materials
Source: J Chem Inf Model. 2023 Jul 18;63(15):4477–82. doi: 10.1021/acs.jcim.3c00217 (PMC10428210; doi:10.1021/acs.jcim.3c00217)
Supplement: Supplementary file 1 — ci3c00217_si_001.pdf [file ci3c00217_si_001.pdf]

Supporting information

PEARS: A web tool for fitting time-resolved photoluminescence decays of perovskite materials

Emmanuel V. Péan[1] and Matthew L. Davies[1,2]

[1]SPECIFIC IKC, Materials Research Centre, College of Engineering, Swansea University Bay Campus, Fabian Way, Swansea SA1 8EN, UK
[2]School of Chemistry and Physics, University of KwaZulu-Natal, Durban, RSA
Corresponding author: m.l.davies@swansea.ac.uk

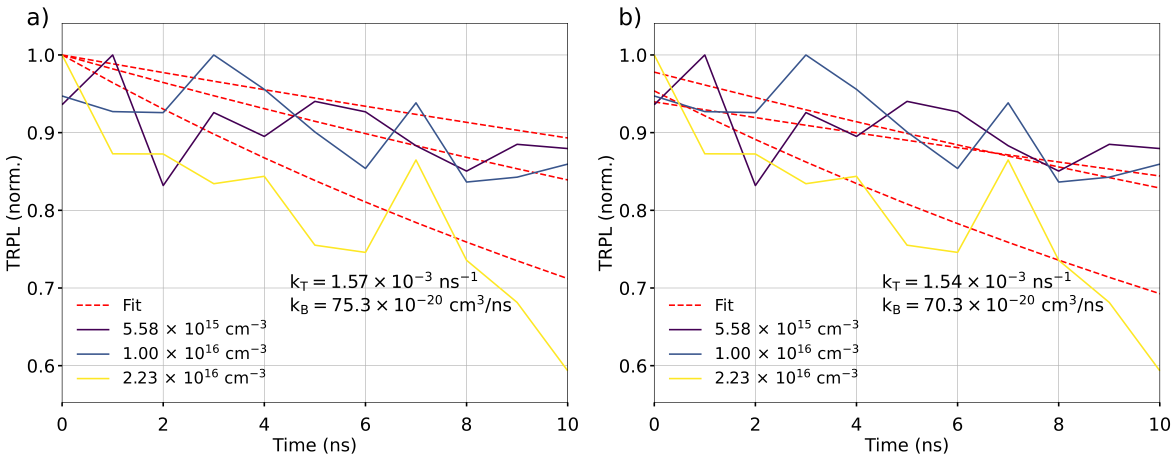

Figure S1: Fitting simulated $(k_T = 1.54 \times 10^{-3}\ ns^{-1},\ k_B = 70.0 \times 10^{-20}\ cm^3/ns)$ TRPL decays with added noise using **a)** a fixed intensity factor $I_0 = 1$ and **b)** a non-fixed intensity factor which is optimised during the fitting. Due to the high amount of noise at the start of the decay, fixing I_0 to 1 forces the decay to decrease faster, which thus leads to an overestimation of the bimolecular recombination constant. At contrary, allowing the intensity factor to be optimised during the least-square optimisation allows to accurately retrieve the parameters.

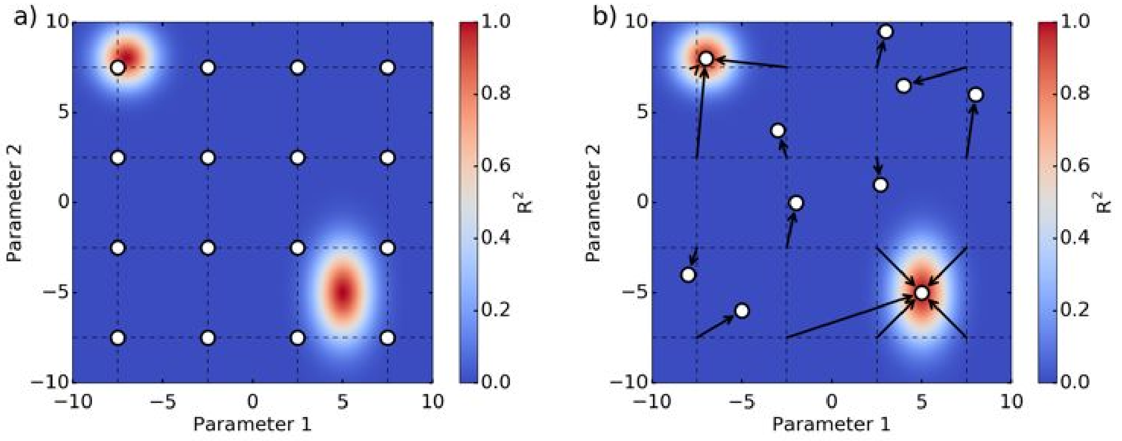

Figure S2: Example of the grid fitting analysis to find local minima based on a model with 2 parameters. The associated R^2 value is displayed in the background to show which parameter values yield a good fit. **a)** A grid of guess values is created as shown by the white dots. **b)** The fitting optimisation is carried out with each set of guess values. Here, 3 and 5 fits converge toward 2 distinct solutions while the remaining optimisations yield incorrect solutions as indicated by their low R^2.

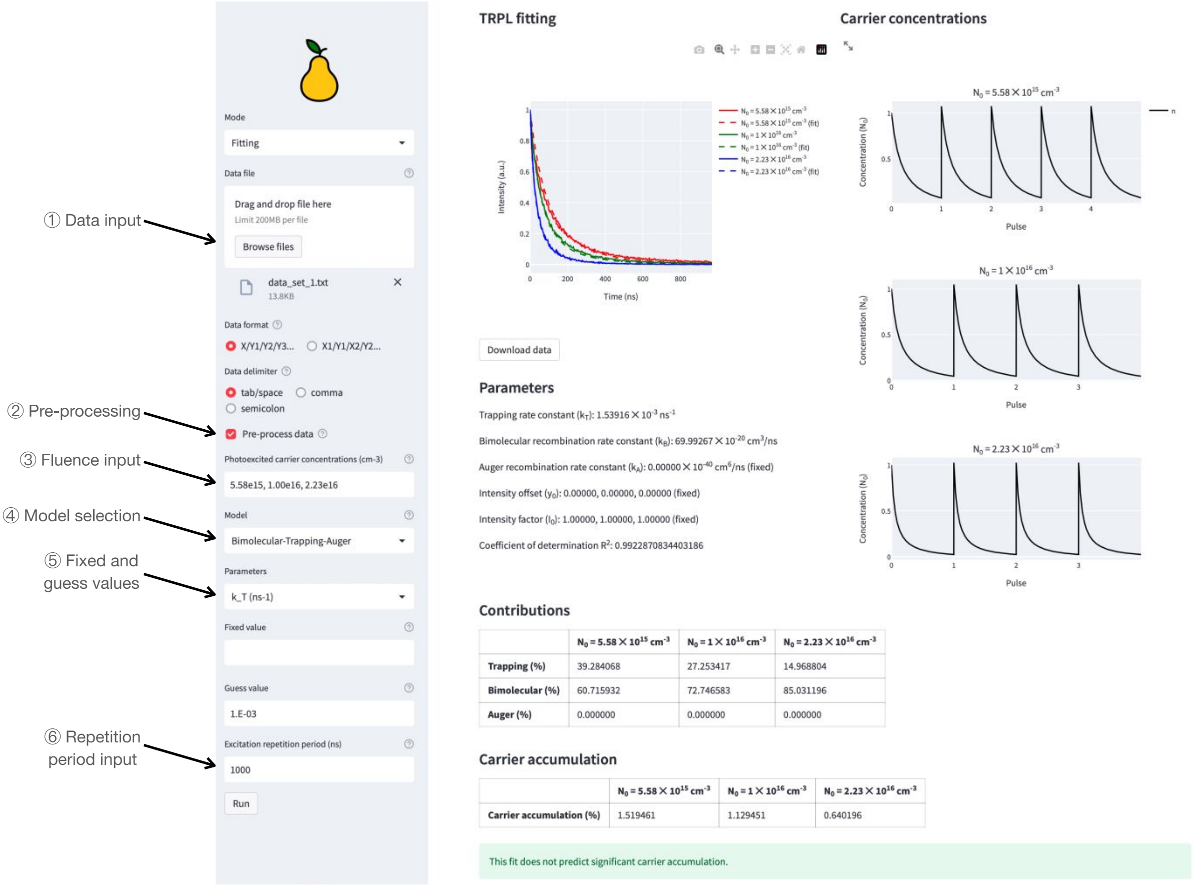

Figure S3: Screenshot of PEARS showing the results of fitting the first data set provided in the "Getting started" section.

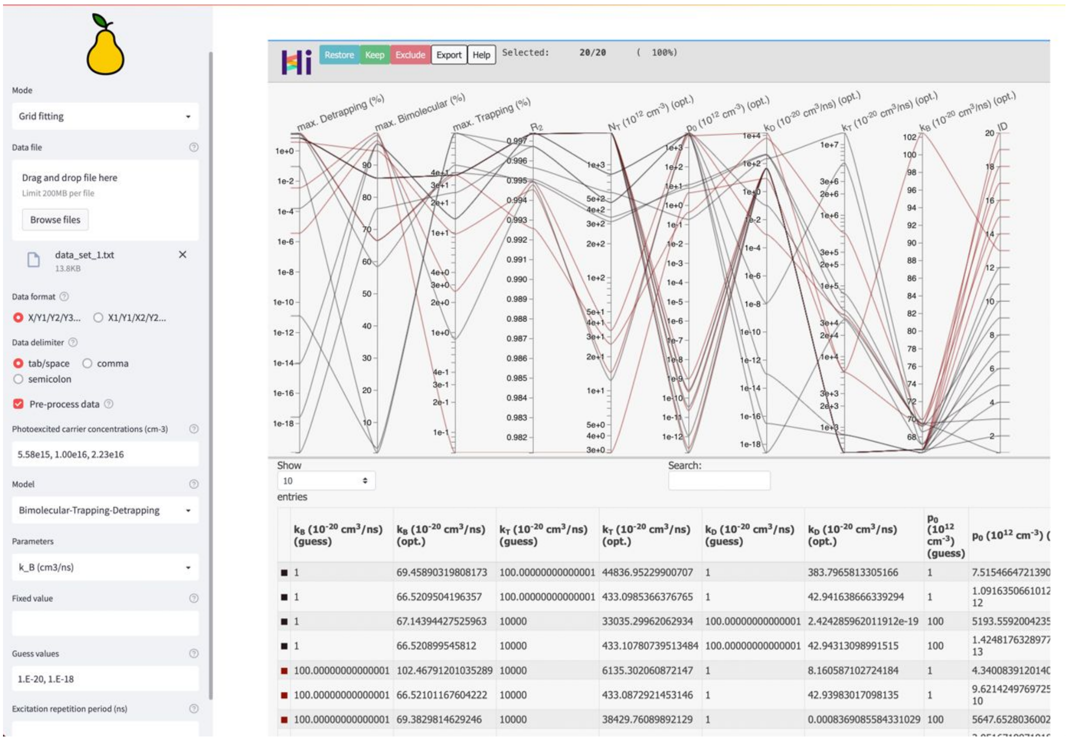

Figure S4: Screenshot of PEARS showing the result of a grid fit using the Bimolecular-Trapping-Detrapping model.
